# Blue and UV LED-induced fluorescence in cotton foreign matter

**DOI:** 10.1186/1754-1611-8-29

**Published:** 2014-12-01

**Authors:** Adnan Mustafic, Changying Li, Mark Haidekker

**Affiliations:** College of Engineering, University of Georgia, 712F Boyd Graduate Research and Studies Center, 30602 Athens, GA USA; College of Engineering, University of Georgia, Driftmier Engineering Center, 30602 Athens, GA USA

**Keywords:** Cotton foreign matter, Fluorescence, Spectroscopy, Blue LED, UV LED, Imaging

## Abstract

**Background:**

Cotton is an important domesticated fiber used to manufacture a variety of products and industrial goods. During harvesting with cotton strippers and cotton pickers, it is contaminated with foreign matter from botanical and non-botanical sources which adversely affect the quality and consistency of cotton, and therefore reduces its market value. To improve the current grading done by the High Volume Instrument (HVI) and human inspectors, it was explored whether fluorescence imaging can be used for cotton foreign matter detection.

**Results:**

Eight types of botanical foreign matter (bark, bract, brown leaf, green leaf, hull, seed coat, seed, stem), and four types of non-botanical foreign matter (paper, twine, plastic bale packaging, plastic bag) were subjected to a fluorescence spectroscopy analysis to determine their optimal excitation and emission wavelength range. Matrix 3D scans were performed in the excitation range from 300 nm to 500 nm, and emission range from 320 nm to 700 nm, and the results indicated the photo-excitable fluorescence in the aforementioned excitation range for all the selected foreign matter categories. Consequently, the blue and the UV LEDs were selected as the excitation sources. The blue LED light provided optimal excitation light for bark, brown leaf, bract, green leaf, hull, and stem, while the UV LED light provided optimal excitation light for paper, plastic bag, plastic packaging, seed, seed coat, and twine.

**Conclusions:**

UV and blue light induces fluorescence in 12 types of botanical and non-botanical cotton foreign matter. An imaging apparatus with blue and UV light excitation sources, and a consumer grade SLR camera was successfully developed to capture and characterize fluorescent images of cotton foreign matter. Based on the results, fluorescent imaging could be a promising method for cotton foreign matter detection. Future studies will focus on the classification of cotton foreign matter categories and to further refine the image processing sequence.

**Electronic supplementary material:**

The online version of this article (doi:10.1186/1754-1611-8-29) contains supplementary material, which is available to authorized users.

## Background

Cotton is the most important natural fiber in the world. Worldwide in 2004, 40% of fiber utilized was cotton, with end products including clothes, home furnishings, industrial products, and food [1]. Food from cotton byproducts includes cottonseed oil, while seed and hull are used to produce animal feed. In the United States, cotton is harvested with cotton pickers and strippers, while in the rest of the world, cotton is primarily harvested by manual labor [1]. One challenge is the introduction of foreign matter to the harvested seed cotton, which is picked up both by human pickers and harvesting machines. The foreign matter, considered to be trash, can be separated into two groups based on their respective origin: botanical and non-botanical foreign matter. Botanical foreign matter (e.g. bark, bract, brown leaf, green leaf, hull, seed coat, seed, stem) originates from cotton plants or other nearby plants, while non-botanical foreign matter is typically brought to the field from elsewhere. The amount of foreign material in cotton is one of the most important cotton quality parameters, even more so when the cotton-processing equipment becomes more automatic. The presence of foreign matter in cotton severely affects cotton grade and thus the price per bale paid by the spinner to cotton growers [2]. In addition, the foreign matter in cotton also reduces the efficiency of the spinning and ginning operations, and eventually lowers the quality of the final woven product. Therefore, rapid identification and classification of the foreign matter in cotton at each stage of cleaning and processing is important to eliminate or reduce the presence of trash and improve ginning/spinning efficiency and quality. Currently, cotton foreign matter grading is performed by human classers and instruments, such as the High Volume Instrument (HVI). Grades assigned by human classers are subjective, and more accurate grading is performed by the grading instruments. The HVI utilizes the geometric method to acquire images of the surface of cotton samples, and it estimates the area occupied by foreign matter, but lacks specificity as to the type of foreign matter detected [3].

Typically, spectroscopic and imaging methods have been used to detect and differentiate cotton foreign matter. A study by Himmelsbach et al. used Fourier Transform-Infrared (FT-IR) spectroscopy combined with Attenuated Total Reflectance (ATR) to retrieve the spectra of distinct cotton foreign matter categories from botanical and non-botanical sources, and compare it to the spectra in the reference database [4]. Spectral matching of reference spectra and sample spectra was observed between Pima cotton foreign matter and Upland cotton foreign matter. The accuracy diminished when samples consisted of a mixture of cotton varieties, and a mixture of experimental varieties, thus necessitating a continuous update of the reference database spectralcontent.

Fortier et al. used FT-Near-Infrared (FT-NIR) spectroscopy to obtain spectra from four botanical cotton foreign matter categories (hull, leaf, seed coat, stem) and compare them to the spectra from the reference database [5]. The results show a 97% foreign matter category prediction accuracy with those found in the reference set, but it needs to be renewed periodically with the reference spectra from new cultivars. A study by the same research group acquired the unique spectral signatures of botanical (hull, leaf, seed, seed coat, stem) and non-botanical (various types of polyethylene and polypropylene plastics) foreign matter in the wavelength range from 800–2500 nm [6]. The aforementioned spectra were compared to the spectra present in the NIR spectral database, resulting in the overall accuracy of 98%. The results could be further improved with the addition of spectra from other types of foreign matter.

Liu et al. attempted classification of seven leaf categories of cotton with a research grade spectrometer [7]. Classification models were developed by employing the soft independent modeling of class analogy (SIMCA) and principal component analysis (PCA). Subsequent application on 650 cotton lint samples with different leaf grades resulted in classification rates ranging from 86% for the spectral region of 405–1095 nm to 95% for the spectral region of 1105–1700 nm. However, the study did not classify different types of botanical foreign matter.

Gamble et al. focused on utilizing fluorescence spectroscopy to determine whether it is possible to quantitatively estimate cotton foreign matter based on their chemical and spectroscopic properties [8]. Extracts of six types of foreign matter (bract, hull, leaf, seed coat, shale, stem) in dimethyl sulfoxide (DMSO) were excited at a single wavelength, producing emission spectra interposed with fluorescence spectra from other foreign matter categories. Results from the partial least-squares (PLS) analysis differentiated hull and leaf due to the presence of a relatively strong emission peak (, ), but the prediction results for bract, seed coat, shale, and stem were poor (, , , ).

Compared to the spectroscopy method, the imaging approach provides the information related to color, geometry, and the spatial distribution of the foreign matter. Xu et al. constructed a cotton trash and cotton measurement (CTCM) system by assembling a video imaging system with the ability to estimate foreign matter content and color parameters of cotton lint samples, but without the ability to differentiate various types of foreign matter [9]. The results of the CTCM were compared to the USDA standards for conventional colorimeters and human classers [10]. High correlations (R^2^ = 0.945–0.999) were reported for color measurements from the CTCM system and those from the HVI, Minolta CR-210 colorimeter, and the grades assigned by human classers. In a later study, the CTCM system was applied to cotton foreign matter differentiation. Xu et al. examined color features of four common types of foreign matter: bark, leaf, inner side of seed coat, and hairy side of seed coat [11]. For this purpose 400 cotton foreign matter particles from 12 cotton sample were analyzed, and their respective color features used as inputs to three clustering methods (sum of squares, fuzzy clustering, and neural networks). Sum of squares clustering and fuzzy clustering yielded accurate foreign matter classification of 83% for leaf category and 93% for bark category, and neural networks had at least 95% accurate classification rate for all foreign matter categories.

Recently, a system with white light and Ultraviolet (UV) excitation light has shown fluorescence imaging could be a useful tool in cotton foreign matter detection. Zhou et al. constructed a system with the capacity to alternate white light and UV excitation light with the goal of foreign matter detection in the spectral range of 250–850 nm [12]. A fluorescence spectroscopy study examined optimal excitation and emission spectral range (excitation range = 320–400 nm, emission range = 420–600 nm) of a single sample of lint containing five foreign matter categories (white bundle strip, black hair, red bundle strip, white paper strip, and white woven strip), and indicated the presence of photoexcitable fluorophores. Preliminary results showed fluorescence imaging could possibly be used to detect both white and colored foreign matter. However, the study presented one sample and it only examined non-botanical cotton foreign matter, whereas the majority of the foreign matter in the US cotton is botanical foreign matter.

Cotton foreign matter identification and differentiation methods so far have either considered a limited number of samples or types of foreign matter, and mainly used spectroscopy and color imaging. Hence, there exists a gap when it comes to the consideration of additional types of imaging modalities like fluorescence imaging to cotton foreign matter detection and differentiation, and a larger number of foreign matter categories need to be taken into account.

To fill the knowledge gap, the overall goal of the study was to conduct an in-depth study on fluorescence imaging of both botanical and non-botanical cotton foreign matter detection. Specific objectives were to:

1. explore whether various cotton foreign matter categories contain fluorophores with unique spectral signatures
2. design a fluorescent imaging system and acquire images of cotton foreign matter
3. establish a qualitative criteria to separate cotton foreign matter from lint.

## Results

### Fluorescence spectroscopy analysis

**Figure 1:**
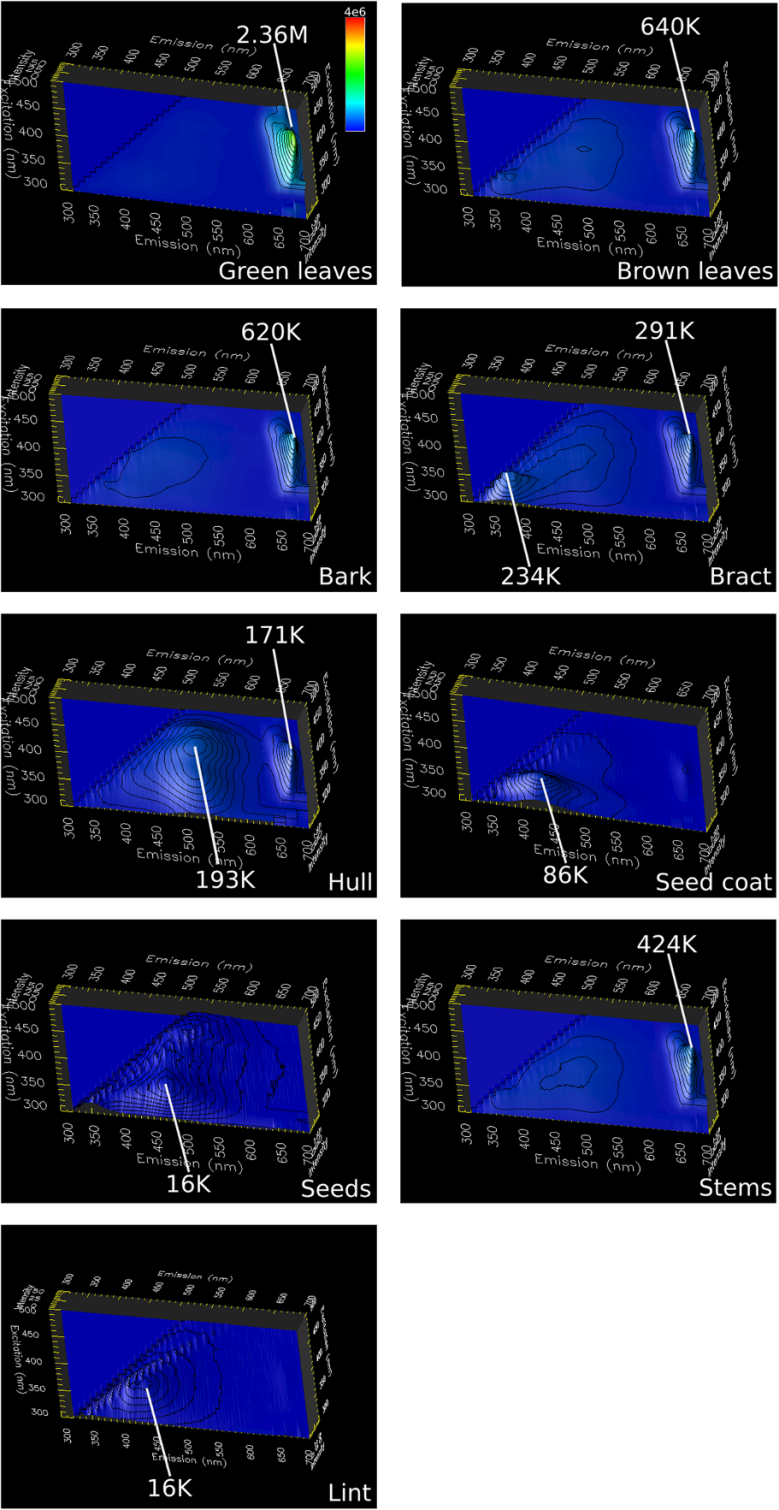
**Matrix 3D scans of botanical foreign matter and lint.** Five foreign matter categories (bark, brown leaf, bract, green leaf, stem) have the optimal excitation wavelength either at 410 nm or 430 nm. Their optimal emission wavelength ranges from 671 nm to 675 nm, and is due to chlorophyll presence. Due to the absence of chlorophyll, seed and seed coat are optimally excited in the UV range (340 nm and 330 nm) with optimal emission at 461 nm and 415 nm, respectively. Hull optimum excitation and emission wavelength is at 400 nm and 516 nm, and lint’s at 360 nm and 434 nm.

Out of eight botanical foreign matter categories, five (bark, bract, green leaf, brown leaf, stem) had their optimal emission peaks in the red region of the visible spectra ranging from 671 nm to 675 nm, with optimal excitation wavelengths either at 410 nm or 430 nm (Figure 1). The red fluorescence in the aforementioned emission range is due to the presence of chlorophyll. Consequently, the strongest measured emission signal was recorded for green leaves at the peak emission of 675 nm (2.38×10^6^ counts/second (cps)), approximately 148 times stronger than the weakest signal recorded for seed at the emission peak of 461 nm (16,000 cps).

Seed and seed coat do not have chlorophyll, and therefore no emission in the red spectral range, but are rather optimally excited at 340 nm and 330 nm, with associated blue emission at 461 nm and 415 nm, respectively. Hull optimal excitation and emission wavelength is at 400 nm and 516 nm, and lint is at 360 nm and 434 nm.

**Figure 2:**
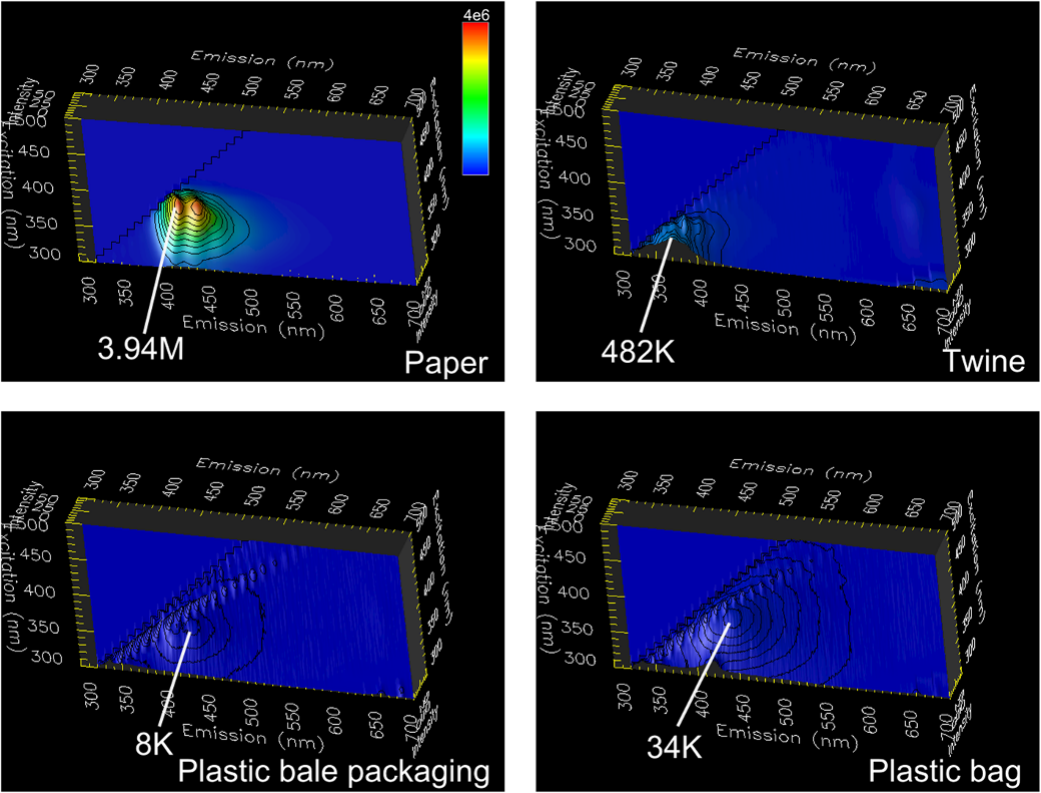
**Matrix 3D scans of non-botanical foreign matter.** Non-botanical foreign matter categories have their optimal excitation wavelengths in the UV range, with paper and plastic bale packaging at 360 nm, plastic bag at 370 nm, and twine at 300 nm. Twine optimal emission is also in the UV range (356 nm), while those of paper, plastic bale packaging, and plastic bag are in the visible light range at 412, 434, and 417 nm, respectively.

Regarding the non-botanical foreign matter, paper had the strongest emission peak at 3.9×10^6^ cps, with optimal excitation and emission wavelength at 360 nm and 412 nm (Figure 2). Plastic bale packaging had the same optimal excitation wavelength, but different optimal emission at 434 nm. Plastic bag was optimally excited at 370 nm, with emission peak at 417 nm. Both plastic bale packaging and plastic bag were weakly fluorescent (8,000 and 34,000 cps). On the other hand, twine had both optimal excitation and emission wavelength in the UV range (300 nm and 356 nm) with peak intensity at 482,000 cps. Lint was found to be optimally excited in the UV range at 360 nm, and optimum blue emission at 434 nm.

From the summary of cotton foreign matter categories and lint, their respective optimal excitation and emission peaks, and peak intensities, it can be observed that five botanical foreign matter categories (green leaf, bark, brown leaf, bract, stem) can be excited in the blue spectral range (410 nm and 430 nm) and emit in the red spectral range (671 nm to 675 nm), with hull optimal excitation at 400 nm and optimal emission at 516 nm (Table 1). Two botanical foreign matter categories (seed, seed coat) can be excited in the UV range (330 nm and 340 nm), similar to all four types of non-botanical foreign matter categories, which have optimal excitation wavelengths in the region from 300 nm to 370 nm. With the exception of twine emitting in the UV region, all foreign matter categories excited in the UV region have their optimal emission peaks in the visible light region.

**Table 1:** **Optimal excitation and emission wavelength, and their associated intensities for botanical foreign matter, non-botanical foreign matter, and lint**

Category	Optimal	Optimal	Intensity
	excitation ***λ***	emission ***λ***	(×10 ^3^ )
	(nm)	(nm)	(cps)
Bark	430	672	620
Bract	430	672	291
Brown Leaf	430	672	640
Green Leaf	410	675	2,360
Hull	400	516	193
Seed	340	461	16
Seed Coat	330	415	86
Stem	430	671	424
Paper	360	412	3,940
Plastic Bag	370	417	34
Plastic Bale Packaging	360	434	8
Twine	300	356	482
Lint	360	434	16

### Fluorescent imaging

**Figure 3:**
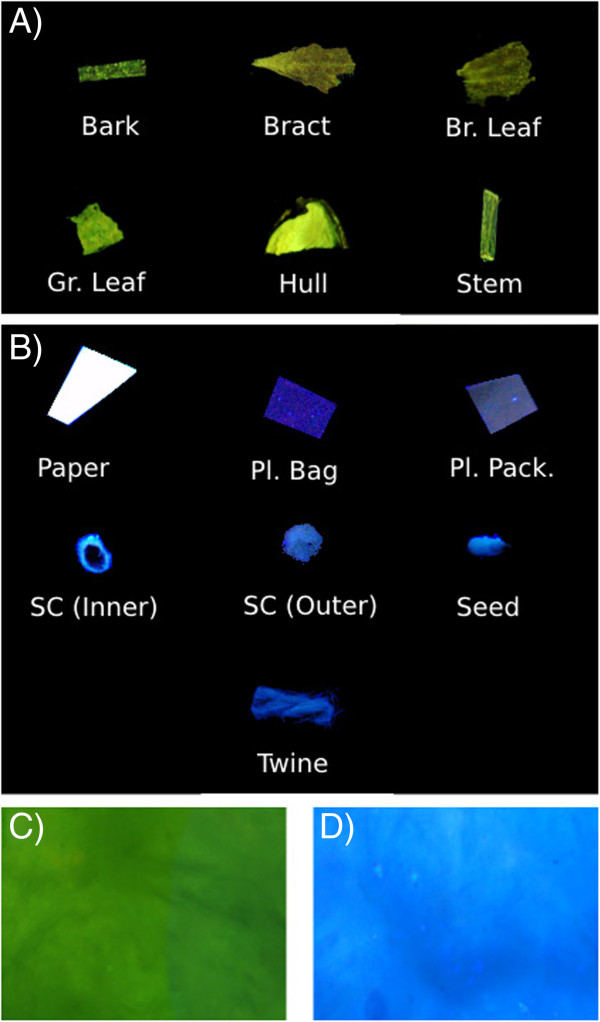
**A total of 13 types of cotton foreign matter and lint illuminated with blue LED and UV LED light.** **A)** 6 types of cotton foreign matter illuminated with UV LED light. **B)** 7 types of cotton foreign matter illuminated with UV LED light. **C)** Lint illuminated with blue LED light. **D)** Lint illuminated with UV LED light.

Green fluorescence emission is dominant in all six types of cotton foreign matter (bark, bract, brown leaf, green leaf, hull, stem) and lint excited under blue LED illumination (Figure 3). To a lesser extent, red fluorescence is also present, but in a secondary role. On the other hand, in all types of cotton foreign matter (paper, plastic bag, plastic packaging, seed, seed coat (inner and outer), twine) and lint excited under UV LED illumination, blue fluorescence emission predominates. Paper is a unique case, and because all of its three channels (red, green, blue) are high in value, it appears white.

**Figure 4:**
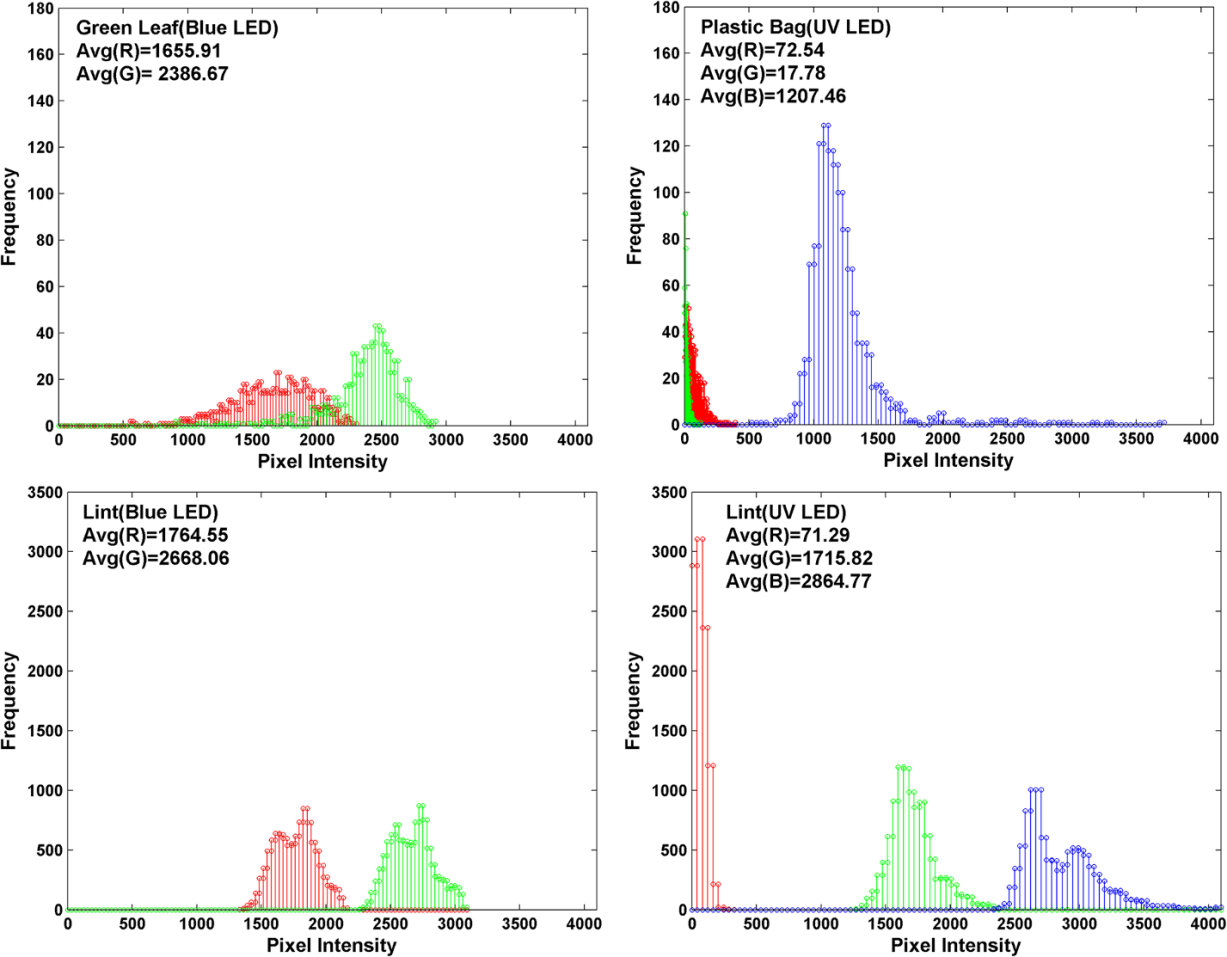
**Examples of histograms of cotton foreign matter and lint.** Two categories of cotton foreign matter shown are green leaf and plastic bag, in addition to lint (for others see Additional files 1, 2 and 3).

If any potential classification of cotton foreign matter is to be done, the first step in the image processing sequence is to distinguish foreign matter from lint. By looking at their respective histograms (Figure 4 and Additional files 1, 2 and 3), cotton foreign matter and lint have varying levels of fluorescence emission. Based on those differences, thresholding criteria specific to particular types of foreign matter can be established.When comparing green leaf and plastic bag to lint, several observations can be made (Figure 4). In the case of green leaf, its red and green channel intensity is lower when compared to the same color channels of lint. When plastic bag is compared to lint, their respective red channels are very close in value. On the other hand, larger difference is found in the green and blue channel where lint exhibited much higher green and blue fluorescence emission.

In general, studies focused on detection and differentiation of cotton foreign matter have typically used white light as a source of illumination. To show how fluorescence emission differs from white light reflectance, a comparison can be made (Table 2). For six types of botanical cotton foreign matter (bark, bract, brown leaf, green leaf, hull, stem) imaged under the blue LED excitation light and white light, lint in general (with the exception of hull) has higher pixel intensity values in all three channels. A similar pattern can be observed for cotton foreign matter imaged under the UV LED excitation light and white light (seed coat, seed, twine), where lint is dominant in most color channels. Paper has higher signal in all three color channels in comparison to lint. Lint exhibited stronger blue and green fluorescence emission than plastic bag and plastic packaging, respectively. However, when imaged under white light, lint has stronger red and green reflectance intensity than plastic bag, and stronger blue reflectance than plastic packaging.

**Table 2:** **Average intensity of lint compared to cotton foreign matter categories with respect to the individual color channels from images under blue LED, UV LED, and white light illumination**

Category	Blue LED	UV LED	White light
Bark, Bract,	Lint has higher	-	Lint has higher red, green,
Brown leaf,	red and green	-	and blue reflectance
Green leaf,	fl. emission	-	
Stem		-	
Hull	Lint has higher	-	Lint has higher red, green,
	green fl.	-	and blue reflectance
	emission	-	
Paper	-	Lint has lower red, green	Lint has lower red, green
	-	and blue fl. emission	and blue reflectance
Plastic bag	-	Lint has higher green and	Lint has higher red and
	-	blue fl. emission	green reflectance
Plastic bale	-	Lint has higher green and	Lint has higher blue
Packaging	-	blue fl. emission	reflectance
Seed coat (Inner),	-	Lint has higher green and	Lint has higher red, green,
Seed coat (Outer),	-	blue fl. emission	and blue reflectance
Seed, Twine	-		

## Discussion

According to the spectroscopic analysis results, five out of eight botanical foreign matter categories exhibited red fluorescence emission in the range from 671 nm to 675 nm. Fluorescence emission in the particular wavelength range can be attributed to the presence of chlorophyll, a green pigment typically found in chloroplasts of green plants, and the leaf mesophyll cells [13]. In contrast, fluorescent images of bark, bract, brown leaf, and stem exhibited mainly green fluorescence. Specifically, we observed fluorescence primarily in the blue-green range, at *λ*_*em*_=461 nm for seeds and *λ*_*em*_=415 nm for seed coat, and hull emitted in the green emission range (*λ*_*em*_=516 nm). In some cases, such as brown leaf and stems, chlorophyll fluorescence diminishes over time as the chloroplasts degrade and the green fluorophores become more prominent [14]. In other cases (i.e., bark, bract, stem), the absence of chlorophyll emission is related to the absence or low density of chloroplasts, making the green fluorescence component prominently detectable. The source for the blue-green fluorescence emission (400 nm to 560 nm) in foreign matter categories originating from the cotton plant can be traced back to the several UV-excitable fluorescing pigments, namely ferulic acid, cinnamic acid, and flavonoids [14]. Ferulic acid is derived from cinnamic acid, and both are found in cell walls, while flavonoid is found in plant vacuoles [15]. In contrast, research by Gamble et al. showed the strongest emission for hull, followed by bract, leaf, and seed coat, and lowest for stem [8]. The aforementioned study used only single excitation wavelength at which the emission spectra of cotton foreign matter (with the exception of hull and leaf) overlapped significantly.

Of the four non-botanical foreign matter categories subjected to fluoroscopic analysis, paper had the strongest emission signal in the blue range (*λ*_*em*_=412 nm). The strong signal can be attributed to the presence of fluorescent whitening agents used during chemical processing of wood pulp in order to alter its color. Twine is spun from fibers made from jute, a plant commonly used to make rope and cloth for industrial use. Jute fibers emit fluorescence under excitation due to the pigments and lignin found in the cell walls [16]. Two other non-botanical foreign matter categories, plastic bag and plastic bale packaging, exhibit fluorescence emission in the blue range (*λ*_*em*_=417 nm and 434 nm) due to the presence of coloring pigments [17]. It should be noted all non-botanical foreign matter categories achieved better optimum emission under the UV LED light, and with the exception of seed and seed coat, botanical foreign matter categories containing chlorophyll achieved better optimum emission under the blue LED light. Since cotton foreign matter is typically found admixed with lint, it is necessary to consider its optimal blue emission band so foreign matter can be segmented out.

Since different types of foreign matter have varying fluorescence emission bands, it is advantageous to look at different channels extracted from fluorescence images. Botanical cotton foreign matter possesses large amount of chlorophyll, thus causing them to emit red fluorescence. However, the red fluorescence changes over time, as cotton plant parts age after they are harvested. The change is typically not uniform, and depends on the cellular process following cell death which determine its degeneration. In this instance, it could be beneficial to focus on the green fluorescence emission caused by additional pigments found in cotton plant (ferulic and cinnamic acid, flavonoids) [14]. Botanical foreign matter categories (seed and seed coat), and non-botanical foreign matter categories (twine, plastic bag, and plastic packaging) do not contain a strong red fluorescence emission, but do contain other fluorescence emitting agents in the green and blue spectral region. The focus on red channel of the color images could be beneficial in foreign matter segmentation from lint, and potential classification between botanical and non-botanical cotton foreign matter. Another source of potentially useful information would be to explore other color models like HSV, in which the illumination component can be separated, and as a result, sources of illumination fluctuations minimized. Color measurements are less likely to change unlike geometric features which change due to the fragility of foreign matter particles being broken into smaller pieces [11]. In the same study, a color imaging system capable of detecting and classifying four categories of botanical foreign matter (bark, leaf, and seed coat) based on their color features was developed. In the current study focus was on exploring whether fluorescence imaging can provide fluorescence emission in the visible spectra which can potentially be used for classification of cotton foreign matter in future studies.

A critical step in the detection of foreign matter in images of lint is the segmentation of the image. In this study we used manual thresholding and supervised application of morphological operators, because the main goal of this study is the determination of the additional information gained by fluorescence imaging. Clearly, supervised segmentation is subject to intra- and inter-observer variability, and an unsupervised method would be desirable. In fact, the images contain a large amount of information that can be used for the future development of unsupervised segmentation methods. One useful assumption is spatial connectivity that allows the use of region-based methods. Second, with images obtained under different lighting conditions, multidimensional thresholding is possible. Third, the widely inhomogeneous illumination advertises the application of further image enhancement (specifically, homomorphic filtering) and the application of locally adaptive thresholding. The development of unsupervised segmentation methods will be subject of further study.

Fluoroscopic analysis of samples necessitated the breakdown of samples with a strong solvent in order to dissolve it thoroughly. Therefore, the total amount of fluorophores detected with spectroscopy is much higher than the amount detected in images because fluorescence signal in images is from surface images. Also, the signals in botanical foreign matter can vary over time because cellular degradation lowers the amount of fluorophores gradually. In contrast smaller changes can be expected for non-botanical foreign matter like paper and plastic. In this study, the fluorescence images were acquired one month after the spectroscopy experiment was done because the optimal excitation and emission wavelengths must be first identified from the spectroscopy data before optical imaging system can be set up. This resulted in the difference between the spectroscopic and the imaging results.

## Conclusions

Excitation in the UV and blue light spectral range induced a fluorescence emission signal for each individual foreign matter categories under consideration. For some of them emission peaks overlapped, however their peak intensity varied. Fluorescence spectroscopy results separate cotton foreign matter categories into two groups according to the types of optimal excitation light used to induced optimum fluorescence emission.

The blue LED light provided optimum excitation light for bark, brown leaf, bract, green leaf, hull, and stem, while the UV LED light provided optimum excitation light for paper, plastic bag, plastic packaging, seed, seed coat, and twine. An imaging apparatus containing blue and UV LED excitation sources was integrated to examine the feasibility of using fluorescence imaging to detect cotton foreign matter. The results showed that fluorescent imaging is a promising tool to detect cotton foreign matter on the surface of cotton lint. Future studies will focus on improving the image analysis process and to classify cotton foreign matter categories.

## Methods

### Sample preparation

**Figure 5:**
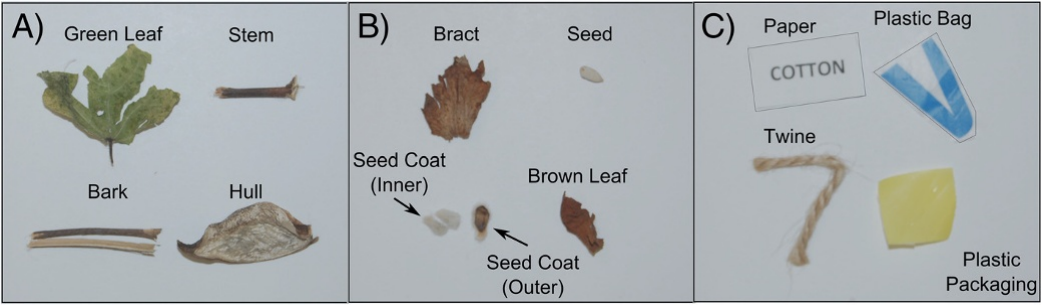
**Cotton foreign matter categories used in this study.** Panels **A** (green leaf, stem, bark, hull) and **B** (bract, seed, seed coat (inner), seed coat (outer), brown leaf) show botanical foreign matter categories, and Panel **C** (paper, plastic bag, twine, plastic bale packaging) shows non-botanical foreign matter categories.

For fluorescence spectroscopy analysis and fluorescence imaging, two types of cotton foreign matter samples were collected and analyzed: botanical and non-botanical foreign matter (Figure 5). Botanical cotton foreign matter samples were collected from seed cotton of four cotton cultivars from the Fall 2012 harvest: Delta Pine 0912, Delta Pine 1050, PhytoGen 499, and FiberMax 1944. Individual botanical foreign matter categories (bark, bract, green leaf, brown leaf, stem, hull, seed, seed coat) were extracted from seed cotton manually, and ground for 90s with an 8000M Mixer/Mill (SPEX SamplePrep, Metuchen, NJ). At the time of analysis, it had been four months since the cotton samples were harvested from the field. Non-botanical foreign matter categories of paper, twine, and plastic bag were purchased from local stores, while plastic bale packaging was obtained from the cotton gin in Tifton, GA, and all were cut into smaller pieces (∼ 2 ×2 mm) with scissors.

### Fluorescence spectroscopy

A total 0.05 g of individual cotton foreign matter categories were extracted with 10 ml of DMSO for 2 hours at room temperature. Extraction was followed by filtering (Whatman 42, 2.5 *μ*m), 2:1 dilution with DMSO, and pipetting 3.5 ml of the solution into a glass cuvette (Starna Cells, Inc., Atascadero, CA), which was then placed into the sample holder of a fluorospectrometer (Fluoromax 3, Horiba, Edison, NJ). Matrix scans were obtained in the excitation range of 300–500 nm, emission range of 320–700 nm, with 0.5 s integration time, 2/2 slits, and figures drawn with OpenDX [18].

### Fluorescent imaging apparatus

**Figure 6:**
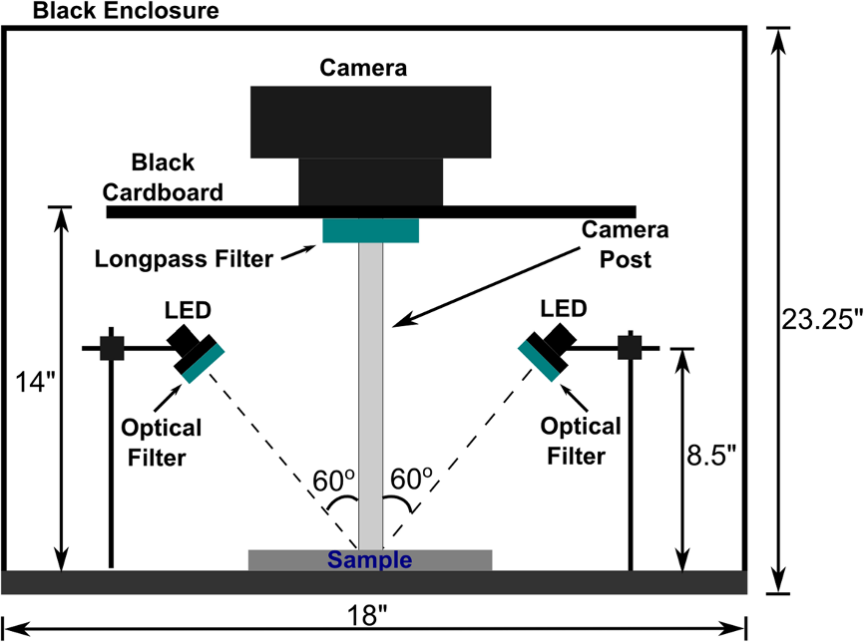
**Side view of the fluorescent imaging apparatus.** Excitation light was provided by two blue LEDs placed at a 60° angle with respect to the camera post in order to homogeneously illuminate the sample under observation, and each equipped with an optical filter in front of it to prevent spectral overlap. An SLR camera with a 500 nm longpass filter was positioned above the sample. The entire apparatus was enclosed in a lightproof enclosure. When other excitation sources were used (UV, white light), the blue LEDs and its bandpass filters were removed, and in the case of UV LED a 400 nm shortpass filter positioned in front of the LED, and a 400 nm longpass filter in front of the SLR camera.

The fluorescent imaging apparatus (Figure 6) consisted of an optical breadboard (Performance Series, 24^′′^×18^′′^×2.4^′′^ (L ×*W*×T), Thorlabs Inc., Newton, NJ), two types of excitation sources used exclusive of each other: two blue LEDs, and a UV LED array. For reference purpose additional white light source was also used. When LEDs (Royal Blue Mounted High Power LEDs, nominal *λ*=455 nm, 1600 mA, Thorlabs Inc., Newton, NJ) were used as excitation sources, light intensity was controlled by two T-cube LED drivers (1200 mA, Thorlabs Inc., Newton, NJ), and power provided by two 15 V power supplies for T-cubes (Thorlabs Inc., Newton, NJ). A bandpass filter (CWL = 450 nm, 40 nm bandpass, Thorlabs Inc., Newton, NJ) was positioned in front of each blue LED to reduce the spectral bandwidth of the emission light. A 500 nm longpass filter (cut-off *λ*=500 nm, Thorlabs Inc., Newton, NJ) was placed in front of the SLR camera to avoid the spectral overlap with the fluorescence excitation of samples under observation.

When the UV LED was used (*λ*=370 nm, Edmund Optics, Barrington, NJ), a 400 nm shortpass filter (Edmund Optics, Barrington, NJ) was placed in front of it, and a 400 nm longpass filter (cut-off *λ*=400 nm, Thorlabs Inc., Newton, NJ) in front of the camera lens instead of the 500 nm longpass filter used in combination with the blue LEDs. When the white light tungsten halogen source was utilized (JCD, GX6503A), the longpass filter positioned in front of the camera was removed, since there was no need to reduce the spectral overlap. Images under white light illumination were used for comparison purposes, while blue LED and UV LED light was used to provide excitation light for fluorescent images.

Cotton samples were centrally positioned on the optical breadboard while the LEDs were mounted on posts adjacent to the sample in order to provide a homogeneous excitation light to the samples under observation. A digital SLR camera (D40, Nikon Inc., Melville, NY) was mounted on a camera stand (Nikon Inc., Melville, NY) vertically above the sample and black cardboard with a hole in the center attached to it block the background fluorescence. The entire fluorescent imaging setup was enclosed and covered with black non-fluorescent cloth to exclude ambient light.

Images were acquired with an f/5.6 aperture and exposure time of 6 s (under blue LED excitation), 1.6 s (under UV light excitation), 1/15 s (under white light illumination), while camera sensitivity was kept at ISO 400. Three different exposure times were used for different types of illumination in order to prevent image saturation. Raw NEF (Nikon Electronic Format) images were converted to 16 bit TIFF images with UFRaw (http://ufraw.sourceforge.net). Images were denoised and binned (4×4) to 759×503 pixels with Crystal Image, a quantitative image analysis software [18]. Further denoising was performed by additional applications of a median and a generalized Gaussian filter.

To compare the red, green, and blue intensities of images taken under the blue LED, UV LED, and white light illumination, five replicates of each type of foreign matter was imaged once under each source of illumination. A fixed, but image-dependent, threshold was used to separate the brightly fluorescent lint from the more weakly-fluorescent foreign-matter regions. This step resulted in a binary mask that contained binary zeros corresponding to lint pixels and binary ones corresponding to foreign mater pixels. In some cases, morphological operations (morphological opening, cluster labeling with a cluster size filter) were applied to the binary mask. The clusters of binary ones effectively acted as regions of interest (ROI) for the foreign matter, thereby effectively separating foreign matter from the lint. The average intensity in the foreign-matter regions was then calculated and compared to the average intensity of the lint layer.

## Electronic supplementary material

Additional file 1: **Figure S1.** Histograms of 13 types of cotton foreign matter and lint imaged under white light. (ZIP 607 KB)

Additional file 2: **Figure S2.** Histograms of 7 types of cotton foreign matter and lint imaged under UV LED light. (ZIP 305 KB)

Additional file 3: **Figure S3.** Histograms of 6 types of cotton foreign matter and lint imaged under blue LED light. (ZIP 366 KB)
